# Ischemic stroke and concomitant gastrointestinal complications- a fatal combination for patient recovery

**DOI:** 10.3389/fimmu.2022.1037330

**Published:** 2022-11-10

**Authors:** Ali A. Tuz, Anja Hasenberg, Dirk M. Hermann, Matthias Gunzer, Vikramjeet Singh

**Affiliations:** ^1^ Institute for Experimental Immunology and Imaging, University Hospital Essen, University of Duisburg-Essen, Essen, Germany; ^2^ Department of Neurology, University Hospital Essen, University of Duisburg-Essen, Essen, Germany; ^3^ Leibniz-Institut für Analytische Wissenschaften - ISAS -e.V., Dortmund, Germany

**Keywords:** stroke, intestinal disturbances, dysbiosis, systemic infections, inflammation

## Abstract

Stroke is primarily a neurodegenerative disease but can also severely impact the functions of other vital organs and deteriorate disease outcomes. A malfunction of the gastrointestinal tract (GIT), commonly observed in stroke patients, is often characterized by severe bowel obstruction, intestinal microbiota changes and inflammation. Over-activated immune cells after stroke are the major contributors to endorse intestinal inflammation and may induce damage to single-layer epithelial cell barriers. The post-stroke leakage of intestinal barriers may allow the translocation and dissemination of resident microflora to systemic organs and cause sepsis. This overshooting systemic immune reaction fuels ongoing inflammation in the degenerating brain and slows recovery. Currently, the therapeutic options to treat these GIT-associated anomalies are very limited and further research is required to develop novel treatments. In this mini-review, we first discuss the current knowledge from clinical studies and experimental stroke models that provide strong evidence of the existence of post-stroke GIT complications. Then, we review the literature regarding novel therapeutic approaches that might help to maintain GIT homeostasis and improve neurological outcomes in stroke patients.

## Introduction

Stroke is the second most frequent cause of death and the third most prevalent cause of human disability in the world. Due to improvements in post-stroke handling including recanalization therapies and prevention studies, the mortality and prevalence of stroke have decreased in the last decades (1). Nevertheless, there is a shift in the overall burden of stroke toward younger ages showing the importance of stroke management and long-term rehabilitation for all adult age groups (2). Even though there are different epidemiological trends among countries, the absolute mortality of stroke patients is expected to increase in the future with one key reason being the longer life expectancy and growing populations worldwide (1, 3). The prediction that elderly people are more susceptible to post-stroke complications suggests the importance and urgency of efficient and targeted treatments. In addition to neurological deficits, stroke patients also show a wide spectrum of non-neurological complications like gastrointestinal (GIT) disturbances, immunosuppression, and bacterial infections in all recovery periods from hyper-acute to chronic phases (1), (**Table 1**). This imposes a significant burden on patients’ overall health and considerably slows post-stroke brain recovery. Moreover, GIT complications are the most frequent cause of increased morbidity and mortality in patients (8). The post-stroke deterioration of intestinal epithelial barriers may allow the invasion of lumen bacteria to systemic tissues and induce activation of immune cells (16). The severity of GIT problems depends on the magnitude of stroke and clinical characteristics of patients, hence, timely prognosis and management of intestinal disorder may reduce systemic and parenchymal inflammation.

**Table 1:** Stroke induces gastrointestinal disturbances in human patients.

Study	Study design	Outcomes
Arnold, M. et al. PloS One, vol. 11,2 (2016) (4)	Evaluation of acute ischemic stroke patients in a tertiary stroke center (Patients=570). Hospital admission evaluation and outcome after three months were analyzed.	Dysphagia was diagnosed in 20.7% of patients and persisted in 50.9% at hospital discharge. Patients with dysphagia suffered more frequently from pneumonia, had longer hospital stays, worse prognoses and higher mortality.
Bonkhoff, AK. et al. J. Am Heart Assoc., vol 11,6 (2022) (5)	Retrospective analysis of German registry data from ischemic stroke patients obtained in 2016 and 2017 (N=152710).	Dysphagia was observed in 22% of stroke patients.
Rofes, L. et al. Neurogastroenterol. Motil (2018) (6).	Prospective longitudinal analysis of patients after hospital admission for up to 12 months (Patients=395).	Oropharyngeal dysphagia was observed in 45% of patients and was an independent risk factor for poor functional outcomes and mortality.
Li, J. et al. Medicine, vol. 96, 25 (2017) (7)	Meta-analysis of clinical studies reporting on stroke patients (Patients=1385).	Constipation incidence was 48%. The frequency was higher in hemorrhagic stroke. Patients in the rehabilitation phase experienced bowel disturbances more frequently than in the acute phase.
Fu, J. et al. Medicine, vol. 98, 28 (2019) (8)	Retrospective analysis of patients with acute cerebral infarction in 2015 and 2016 (Patients= 1662).	GIT bleeding incidence after stroke was 8.4%. One-year mortality was higher in patients with GIT bleeding.
Du, W. et al. Stroke Vasc Neurol. vol. 5, 2 (2020) (9)	Retrospective analysis of patients with ischemic stroke and GIT bleeding in China National Stroke Registry from symptom onset to 12 months (Patients=12415).	GIT bleeding was an independent risk factor for stroke recurrence within 3, 6 and 12 months.
Roth, WH. et al. Stroke (2020) (10)	An exploratory analysis evaluating the relationship between GIT problems and ischemic stroke (Patients=1.725.246).	GIT disorders were associated with an increased risk of future ischemic stroke.
Xia, GH. et al. Front. Neurol., vol 10, 937 (2019) (11)	Fecal microbiota of 104 ischemic stroke patients was characterized using 16S rRNA sequencing and compared with microbiota of healthy subjects (Patients=104, healthy controls=90).	Stroke causes dysbiosis with 18 bacterial genera being significantly different in comparison to healthy subjects. The degree of dysbiosis was associated with increased inflammation and deteriorated stroke outcomes.
Li, N. et al. BMC Microbiology (2019) (12)	Fecal microbiota changes in ischemic stroke patients and healthy controls were analyzed using 16S rRNA sequencing (N=30 per group).	Gut microbiota dysbiosis was observed in stroke patients and their microbiota was enriched with SCFA-producing bacterial genera compared to healthy subjects.
Tan, C. et al. JPEN. vol. 45,3 (2021) (13)	Fecal microbiota analysis using 16S rRNA sequencing. Fecal SCFAs levels were measured by gas chromatography for up to 3 months (Patients=140, healthy controls=92).	Dysbiosis was observed in stroke patients. A lack of SCFA-producing bacteria and reduced SCFAs levels were found in patients compared to controls. Changes were associated with poor stroke outcomes.
Haak, BW. et al. Transl. Stroke Res. 12, 581–592 (2021) (14)	Fecal microbiota changes were analyzed after one day of hospital admission using 16S rRNA amplicon sequencing (Patients=349 and healthy controls=51).	Gut microbiota was severely altered after ischemic and hemorrhagic stroke compared to healthy controls. Enrichment of TMAO-producing and loss of butyrate-producing bacteria after stroke.
Xu, K. et al. Gut (2021) (15)	Fecal microbiota analysis of stroke patients using 16s rRNA amplicon sequencing (Patients=152 and healthy controls=28).	Stroke-induced dysbiosis with the expansion of Enterobacteriaceae and was an independent risk factor for a worse outcome.

## Stroke induces gastrointestinal complications

Several studies have demonstrated GIT disturbances in stroke patients such as dysphagia, gastrointestinal bleeding, or constipation (17). A recent stroke registry study showed that 19.6% of patients experience swallowing problems and indicate dysphagia frequency of 75.4% which is associated with a high risk of death during hospital admissions (5). Approximately 50% of the dysphagia complications persisted even after hospital discharge (4, 6, 17), causing a persistent burden on patients’ health. One of the widely discussed pathophysiological explanations for dysphagia after stroke is cranial nerve involvement, thereby causing severe malnutrition in patients (4). With dysphagia, paralytic ileus causing constipation is the second most prominent symptom observed in patients with brain stem infarcts, with an overall incidence of 45%, of which 30% had proximal colonic transit delay (7, 18). Conversely, in addition to constipation history being related to worse stroke outcomes, the usage of laxatives increases stroke risk in patients with constipation (19). Furthermore, old-age patients with existing disorders of GIT such as dysbiosis, hypertension, diabetes mellitus, and intestinal infections present a higher risk of future ischemic stroke (10). These exploratory data from a large number of patients indicate that even before stroke, gastrointestinal problems might exist and may increase the incidences of stroke. The reason for this derived relationship might be that these complications are also the major comorbidities in cerebrovascular diseases. Previous studies have consistently reported a higher rate of mortality in women patients compared to men patients and was associated with aging, stroke severity, and atrial fibrillation (20, 21). However, the presence of GIT complications did not differ between men and women patients (21). Experimental studies using animal models of stroke have also highlighted the beneficial role of male and female hormones in reducing brain inflammation and injury (22). Conversely, the clinical studies investigating the circulating levels of hormones and their relationship to post-stroke functional outcomes have provided inconsistent results. How GIT complications in patients promote vascular pathology and associated stroke risk are important questions for further research.

**Figure 1:**
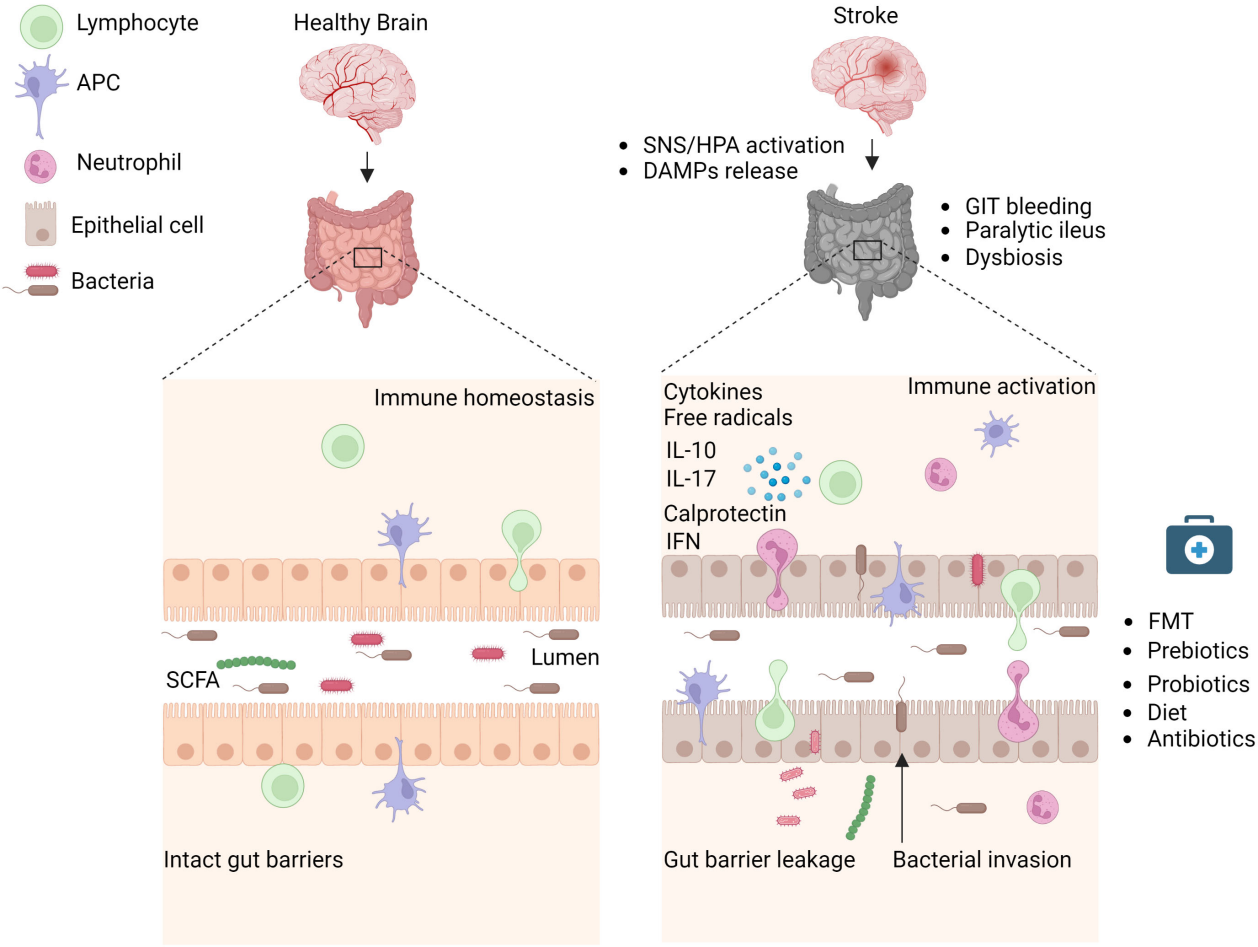
Stroke induces intestinal paralysis, barrier leakage, and inflammation. Left panel: In healthy conditions, there is immune homeostasis at intact intestinal barriers with balanced microbiota. Right panel: After stroke, the activation of SNS/HPA axis and release of DAMPs trigger microbiota dysbiosis, and paralytic ileus and promote immune cell-driven inflammation. Activated neutrophils, macrophages, and dendritic cells release toxic cytokines, free radicals, and proteases and cause epithelial damage. Invasion of intestinal bacteria to systemic tissues enhances immune cell activation and neuroinflammation. Rebalance of dysbiotic microbiota with pre and probiotics, food supplements, or its depletion with antibiotics is associated with reduced brain inflammation and improved stroke outcome in experimental studies and their application to stroke patients requires validation. APC, Antigen-presenting cell, DAMPs, Damage-associated molecular patterns, FMT Fecal microbiota transplantation, HPA, Hypothalamus Pituitary Adrenal; IFN, Interferon; IL, Interleukin; SCFA, Short-chain fatty acid; SNS, Sympathetic Nervous System. Created with BioRender.com.

GIT bleeding is diagnosed in about 0.1-8.4% of stroke patients and contributes to high mortality (8, 23). The use of calcium channel blockers, steroid hormones, or nonsteroidal anti-inflammatory drugs increases the risk of GIT bleeding (8). Of note, when a patient is diagnosed with GIT bleeding, antithrombotic treatment is not endorsed as this can further increase susceptibility to bleeding-related mortality, causing limitations to existing approved therapies (24). GIT bleeding is also an independent risk factor for recurrent stroke, indicating bidirectional crosstalk between the intestine and brain (9). Moreover, patients with large cortical infarcts have more severe gastrointestinal symptoms that significantly correlate with worse outcomes (6, 8). Other than early diagnosis, erythrocyte transfusion, and endoscopic interventions, there are no approved therapies against GIT bleeding, thus requiring careful patient diagnosis in hospitals (24). The recent experimental studies using animal models of stroke have largely reproduced similar patterns of GIT disturbances observed in stroke patients (**Figure 1**). Using a fluorescence gastric bolus tracking approach, we demonstrated that GIT in mice is severely paralyzed after a large stroke (25). A recent work from Ye et al. showed increased intestinal permeability and signatures of local inflammation after experimental stroke (26). Their results demonstrated reduced intestinal motility and lower expression of tight junction proteins in intestinal tissue which closely relates to constipation and intestinal inflammation observed in stroke patients (26). Future studies in animal models of experimental stroke will greatly help to understand the in-depth mechanisms of gastrointestinal changes and the development of targeted therapies.

## Stroke changes intestinal microflora and increases inflammatory responses

The human intestine harbors more than 100 trillion bacteria with *Bacteriodetes* and *Firmicutes* as the most abundant phylotypes (27). The fermentation of dietary fiber by intestinal bacteria generates short-chain fatty acids (SCFAs) such as acetate, propionate, and butyrate which influence intestinal barrier integrity and regulate inflammatory processes (28, 29). Moreover, intestinal microflora can modulate the levels of important neurotransmitters, thus playing a crucial role in nervous system development and physiology (29). Interestingly, most bacterial species in the human GIT are not cultivable but their identification using 16S rRNA sequencing has helped to analyze microbiota changes in different brain diseases. The composition of intestinal microbiota is severely altered (a process termed dysbiosis) in stroke patients and a higher abundance of genera *Enterobacteriaceae* is an independent risk factor for poor outcomes (15). In addition, UniFrac distance analysis revealed altered microbiota structure in the acute and subacute phases of stroke. However, α-diversity was only reduced in the convalescent phase (1-3 months post-stroke) (15). On the contrary, Li et al. showed no differences in microbiota α-diversity between healthy controls and stroke patients that were sampled within two days of admission (12). These results may indicate delayed kinetics of large-scale microbiota changes in stroke patients.

The mechanisms underlying post-stroke alterations in specific bacterial species are still unknown but have been reasoned to be affected by intestinal paralysis and increased inflammation. Intestinal microbiota composition or produced metabolites show a positive correlation with serum inflammatory markers in stroke patients. For instance, *Lactobacillus ruminis* levels in stool were correlated to elevated serum interleukin-6 concentrations, while valeric acid levels were associated with serum C-reactive protein (30). Interestingly, intestinal microbiota of stroke patients is enriched with SCFA-producing bacteria *Odoribacter, Akkermansia, Ruminococcaceae* and *Victivallis* (12). Higher levels of SCFAs in stroke patients who underwent thrombectomy were associated with increased systemic inflammation and poor stroke outcomes (12, 31). However, different studies on analyzing the plasma or fecal levels of SCFAs in stroke patients and underlying outcomes have derived variable results. A new clinical study involving 141 aged stroke patients showed a low abundance of butyrate-producing bacteria and reduced levels of fecal butyrate in the high-risk group compared to the low-risk group (32). In this regard, animal models of stroke have significantly contributed to deciphering the interrelationship between SCFAs and stroke outcomes. A study by Sadler et al. showed that four weeks treatment of mice with SCFAs improves post-stroke cortical reorganization and increases neuronal spine density (33). The beneficial effects were further correlated with reduced microglia activation and brain invasion of T lymphocytes. These results are in line with previous research findings demonstrating the impact of microbiota-derived SCFAs on microglia functions (34, 35). The analysis of post-stroke changes in intestinal microbiota or their produced molecules may help to predict stroke severity. Recently, Xia et al. performed 16S rRNA analysis on patients’ fecal samples and generated a Stroke Dysbiosis Index (SDI), demonstrating that a higher SDI was associated with larger brain infarcts and high mortality (11). The SDI established in this study might help to predict clinical prognosis after stroke. Another study showed that the bacterial metabolite-trimethylamine N-oxide (TMAO) is increased after stroke and correlates with recurrent cardiovascular events (36). Those prognostic factors might shed further light on clinical paradigms to closely monitor patients with cardiovascular disease.

Inflammation is a crucial part of stroke-related GIT complications. In healthy conditions, intestinal homeostasis is maintained between immune cells, intestinal microflora and epithelial cells. In inflammatory disorders, activated macrophages release chemokines that attract neutrophils to the intestinal lamina propria (37). The activated neutrophils release toxic cytokines, free radicals, and proteases, causing further disruption of a single-layer intestinal epithelial cell barrier. On the other side, neutrophils can phagocytose the luminal bacteria that translocate through the epithelial barriers and may reduce systemic infections (38). Intestinal microbiota-generated SCFAs have been shown to regulate the inflammatory functions of neutrophils (39, 40). In addition, neutrophil maturation is regulated by gut microbiota *via* the involvement of toll-like receptors and myeloid differentiation factor 88-mediated signaling pathways (41). However, further research is required to understand the functions of neutrophils in post-stroke intestinal inflammation. Previous studies observed that activated neutrophils can migrate to intestinal tissues and release the inflammatory protein calprotectin (42). Stroke patients show increased levels of fecal calprotectin which positively correlates with high concentrations of serum inflammatory C-reactive protein. Moreover, fecal calprotectin amounts negatively correlate with serum albumin levels and the Glasgow Coma Scale which is suggested as an indicator of intestinal inflammation (43). Thus, activated neutrophils after stroke can increase intestinal inflammation and fecal calprotectin can be a promising biomarker for diagnosing intestinal inflammation.

In the last years, experimental research has largely contributed to our understanding of post-stroke disturbances at the intestinal-brain-immune axis. Recently, we have shown that intestinal microbiota after stroke is severely changed in mice and activates T cells in systemic and intestinal lymphoid tissues (25, 44). This study showed that proinflammatory T cell subsets Th1 and Th17 in intestinal Peyer’s patches can migrate to the injured brain and increase tissue injury *via* the release of toxic cytokines (25). In line with our findings, Benakis et al. observed a lower number of intestinal γδ T cells after experimental stroke in mice that caused reduced secretion of neuroprotective cytokine interleukin-10 (45). T regulatory (Treg) cells exhibit neuroprotective functions after stroke *via* the release of anti-inflammatory cytokines such as IL-10 and TGF-β (46). Intestinal microbiota plays a significant role in the development of Treg cells and microbiota-deficient germ-free mice exhibit low numbers of IL-10-producing Treg cells in lymphoid organs (47). Previous studies have highlighted the contribution of microbiota-derived SCFAs in the generation of Treg cells in intestinal lymphoid tissues (48, 49). But, how intestinal microbiota changes after stroke influence the activation of T cells is still an open question. Nevertheless, recent findings have demonstrated that intestinal bacteria can invade systemic tissues and induce sepsis-like conditions. For example, Stanley et al. showed that intestinal bacteria can invade organs like liver, spleen and lungs in mouse models of experimental stroke (16). In addition, conventional intestinal bacteria such as *Enterococcus* species, *Escherichia coli* and *Morganella morganii* were identified in the sputum of stroke patients with pneumonia (16). In this study, the number of patients was low (N=8) however the high mortality of about 37.5% may relate to severe stroke and related pneumonia (16). Hence, studies with a higher number of stroke patients are required to identify intestinal bacterial species that may induce pneumonia or sepsis-like conditions. A further understanding of the mechanisms of intestinal bacteria invasion and spread would be important to specifically block these routes and inhibit post-stroke inflammation.

## Potential therapies for post-stroke GIT dysfunction

The GIT complications after stroke strongly modify the disease pathogenesis and are associated with an unfavorable functional outcome. Thus, the identification of potential therapeutic approaches to prevent these side effects is a clinical priority. In this regard, the following therapeutic regimens have been clinically explored in stroke and other brain diseases and the results are optimistic, but require careful interpretation in multi-center clinical trials.

## Antibiotics prophylaxis

Post-stroke bacterial infections are a common complication after stroke (17). For its treatment, multiple clinical trials have tested a combination of different antibiotics. A recent multicenter and randomized clinical trial investigated the effect of intravenous ceftriaxone given daily for four days and analyzed the infection rates and functional outcomes at three months (50). The study results showed a reduction in post-stroke infections but no significant improvement in functional outcomes. Two large meta-analysis studies included patients from more than seven clinical trials that received prophylactic antibiotics treatment at the time of stroke onset and found significantly decreased levels of bacterial urinary tract infections but not pneumonia (51, 52). On the contrary, a recent study by Benakis et al. observed neuroprotection in mice that were pre-treated with antibiotics for four weeks (53). However, the results are difficult to anticipate in clinical situations when treating patients in acute or subacute phases after stroke. The absence of antibiotics protective effects in stroke patients can be due to multiple reasons; (a) the acceleration of existing stroke-induced dysbiosis (b) reduced levels of bacterial metabolites involved in epithelial barrier integrity (c) severe immunosuppression in patients and (d) antibiotic resistance in pneumonia-causing bacterial strains. Thus, instead of using antibiotics, cause-aiming treatment modalities that help to maintain intestinal homeostasis should be introduced.

## Healthy microbiota transplantation

Fecal microbiota transplantation (FMT) is the transfer of intestinal bacteria and other microbes from a healthy donor to a patient. This process is performed to restore microbiota and inhibit the detrimental effects of dysbiosis (54). In healthy volunteers, FMT is suggested to be a safe procedure without any long-term complications (55). A recent study has reported the beneficial effect of FMT in a patient with secondary progressive multiple sclerosis and showed disease stability for over ten years (56). Furthermore, FMT is currently the most effective intestinal microbial intervention and approved therapy for frequent *Clostridioides difficile* infections (54). Interestingly, FMT composition enriched in *Bifidobacterium* produced a more favorable symbiosis after transplant and indicates the necessity of microbiota characterization in healthy donor FMT samples to select more favorable bacterial species (57). Considering the beneficial impact of FMT in other neurodegenerative diseases, this approach might prove helpful in stroke patients (58, 59).

## Prebiotics and probiotics

Prebiotics act as a substrate to be used solely by the host microbiota, which has regulatory and balance-maintaining effects. Human milk oligosaccharides, fermentable fibers, and indigestible oligosaccharides are examples of prebiotics. Earlier studies have evidenced the protective function of prebiotics in reducing neuroinflammation and improving cognitive function in Alzheimer’s disease patients (60, 61). The treatment of obese patients with prebiotic inulin was shown to increase plasma acetate and improve fat oxidation (62). Probiotics comprise a combination of microorganisms that have beneficial effects on human health. Most are bacterial strains that can produce lactic acid *via* fermentation such as *Lactobacillus, Bifidobacterium* and *Lactococcus*. A recent meta-analysis by Liu et al. showed that stroke patients treated with enteral nutrition including probiotics had a better outcome and reduced incidence of bacterial infections (63). Similarly, another meta-analysis study involving 26 randomized controlled trials in probiotics-treated stroke patients revealed a significant reduction in GIT complications and lower incidences of bacterial infection (64). Importantly, the composition of probiotic regimens used in different patient studies differs and might have unsolicited effects. Thus, probiotics with standardized molecules and proven beneficial effects in pre-clinical stroke models and multi-center clinical trials would be required for their translation to clinics.

## Dietary interventions

Stroke patients often suffer from dysphagia and require the placement of a nasogastric tube or endoscopic gastrostomy (PEG) (17) which shows positive effects (65, 66). Nutritional support in stroke patients *via* oral feeding is crucial and decreases mortality. A balanced diet supplementation in stroke patients may improve stroke outcomes possibly by reducing intestinal microbiota changes and associated inflammation. The manipulation of diet contents for stroke treatment might serve as a vital therapeutic option but findings in animal models of stroke have provided inconsistent results. For example, the administration of a high-protein diet in rats has been shown to reduce post-stroke neurological deficits (67). In contrast, we recently showed that the restriction of dietary protein from 20% to 8% rebalanced intestinal microbiota, reduced brain inflammation, and was neuroprotective after stroke in mice (68). These variable results might be due to the differences in used animal models or the composition of protein diets. Furthermore, a Mediterranean diet with a higher percentage of plant-based foods and olive oil was shown to have short and long-term protective effects on stroke outcomes (69). Another study demonstrate that feeding stroked mice with a high-fat diet increased infarct volumes and more hemorrhagic complications *via* the mechanisms involving altered lipid profiles and high blood sugar levels (70). Other important nutrients in the human diet are choline and carnitine which are required for the synthesis of acetylcholine, betain, phopholipids, and trimethylamine (TMA). Choline exerts a wide range of beneficial effects like reducing inflammation, placental health, and positive neurocognitive effects. Intestinal microbiota can also convert choline/carnitine into TMA which is then metabolized into trimethylamine N-oxide (TMAO) by host hepatic monooxygenases (71). Despite many protective functions, higher circulating levels of TMAO and its precursors are related to an increased risk of stroke and poor functional outcomes (72, 73). Diets with reduced levels of choline/carnitine or probiotics to reduce TMAO biosynthesis may help to reduce the incidences of cardiovascular events such as atherosclerosis and ischemic stroke (74). Thus, diet manipulation studies after stroke require further standardization and large-scale validation in multicenter preclinical trials before their application to stroke patients.

## Summary and future prospective

There is extensive clinical and experimental evidence that highlights the existence of GIT disturbances after stroke. However, the clinical trials focused on treating stroke-associated intestinal comorbidities are still very limited.

Post-stroke disturbances of GIT can increase bacterial infections and systemic inflammation that reversibly impacts brain tissue degeneration. The clinical trials on the use of antibiotics as prophylaxis treatment have not delivered promising results and suggest research on new avenues to benefit stroke patients. In this direction, recent preclinical studies have shown the protective effects of regimens that focused on rebalancing disturbed intestinal microbiota *via* FMT (25) or diet manipulations (68). As in the emergency stroke treatment motto, “Time is brain”, a new point of approach “Gut is brain” could be proposed to impose the crucial role of intestinal microbiota on post-stroke pathologies and long-term outcomes.

In terms of stroke-induced immunosuppression and the susceptibility of patients toward bacterial infections, there is an urgent need to find the underlying pathomechanisms and treatment options. Our recent findings have highlighted the key role of the stroke-induced release of circulating DNA as a major factor in causing lymphocyte loss in systemic lymphoid tissues (75). However, if stroke can induce a similar loss of lymphocytes in intestinal immune compartments is not completely known. The understanding of these pathways might help to uncover novel drug targets to maintain immune homeostasis at the intestinal barriers and reduce GIT complications. Further research on testing the effects of novel therapeutics for correcting intestinal immune dysbalance will help to reduce post-stroke neuroinflammation and ongoing brain degeneration.

## Author contributions

AT and VS designed and wrote the article. AH, DH, and MG critically revised the article. All authors have approved the submitted version. All authors contributed to the article and approved the submitted version.

## Funding

This research work was funded by the Deutsche Forschungsgemeinschaft (DFG), research grant SI 2650/1-1 to VS, GU 769/10-1 to MG, HE 3173/11-1, 3173/12-1, 3173/13-1 and 3173/15-1 to DMH and the CRC TRR332 (project C6) to MG and DMH.

## Conflict of interest

The authors declare that the research was conducted in the absence of any commercial or financial relationships that could be construed as a potential conflict of interest.

## Publisher’s note

All claims expressed in this article are solely those of the authors and do not necessarily represent those of their affiliated organizations, or those of the publisher, the editors and the reviewers. Any product that may be evaluated in this article, or claim that may be made by its manufacturer, is not guaranteed or endorsed by the publisher.
